# Serum IL-18 Is Closely Associated with Renal Tubulointerstitial Injury and Predicts Renal Prognosis in IgA Nephropathy

**DOI:** 10.1155/2012/728417

**Published:** 2012-02-06

**Authors:** Beili Shi, Zhaohui Ni, Liou Cao, Minjie Zhou, Shan Mou, Qin Wang, Minfang Zhang, Wei Fang, Yucheng Yan, Jiaqi Qian

**Affiliations:** Renal Division, Renji Hospital, Shanghai Jiaotong University School of Medicine, 1630 Dongfang Road, Shanghai 200127, China

## Abstract

*Background*. IgA nephropathy (IgAN) was thought to be benign but recently found it slowly progresses and leads to ESRD eventually. The aim of this research is to investigate the value of serum IL-18 level, a sensitive biomarker for proximal tubule injury, for assessing the histopathological severity and disease progression in IgAN. *Methods*. Serum IL-18 levels in 76 IgAN patients and 36 healthy blood donors were measured by ELISA. We evaluated percentage of global and segmental sclerosis (GSS) and extent of tubulointerstitial damage (TID). The correlations between serum IL-18 levels with clinical, histopathological features and renal prognosis were evaluated. *Results*. The patients were 38.85 ± 10.95 years old, presented with 2.61 (1.43∼4.08) g/day proteinuria. Serum IL-18 levels were significantly elevated in IgAN patients. Baseline serum IL-18 levels were significantly correlated with urinary protein excretion (*r* = 0.494, *P* = 0.002), Scr (*r* = 0.61, *P* < 0.001), and eGFR (*r* = −0.598, *P* < 0.001). TID scores showed a borderline significance with serum IL-18 levels (*r* = 0.355, *P* = 0.05). During follow-up, 26 patients (34.21%) had a declined renal function. Kaplan-Meier analysis found those patients with elevated IL-18 had a significant poor renal outcome (*P* = 0.03), and Cox analysis further confirmed that serum IL-18 levels were an independent predictor of renal prognosis (*β* = 1.98, *P* = 0.003).

## 1. Introduction

IgA nephropathy (IgAN), a mesangial proliferative glomerulonephritis (GN), is the most commonly occurring glomerulonephritis worldwide [1]. It was once thought to be relatively benign and to have a reasonably good long-term prognosis. However, recent studies indicate that IgAN has the potential for slowly progressive chronic renal impairment, leading eventually to ESRD. Approximately 25 to 30% of any published cohort will require renal replacement therapy within 20 to 25 years of presentation. From first symptoms, 1.5% of patients with IgAN have been calculated to reach ESRD per year [2, 3]. Prognostic clinical factors for the future development of renal failure include the presence of persistent and severe proteinuria, elevated serum creatinine (Scr) at diagnosis, and the presence of hypertension; meanwhile, histologically the extent of tubulointerstitial fibrosis correlates better with reduced renal function than glomerular histology does [4]. 

 Interleukin-18 (IL-18) is a member of the IL-1 family of cytokines and was originally described as an interferon gamma (IFN-*γ*) inducing factor [5]. It is a novel biomarker that has been studied in detail in preclinical ischemia-reperfusion models and has been proved to play an important role in renal injury induced by acute ischemia-reperfusion in mice [6]. Numerous reports indicate that CKD patients have elevated serum or urine levels of IL-18 and its correlation with decreased renal function [7–9]. The aim of this research was to investigate the relationship of serum IL-18 levels with histopathological severity and renal prognosis in IgA nephropathy.

 We performed a prospective study using serum IL-18 levels, detailed baseline clinical data, and semiquantitative analysis of renal biopsies, to assess the correlation between IL-18 and tubulo-interstitial damage and the value of IL-18 in determining adverse outcomes, mainly disease progression.

## 2. Materials and Methods

### 2.1. Patients

The study protocol was approved by the Ethics Committee of Shanghai Jiaotong University School of Medicine (Shanghai, China) (2002HL0133). 76 patients were enrolled (39 females) and followed after they gave fully informed consent. Inclusion criteria consisted of (1) biopsy-proven IgA nephropathy, histological grade III or above according to Lee's grading system [10] within a 6-month period, (2) an age of 18 to 65 years, (3) estimated glomerular filtration rate (eGFR) greater than 30 mL/min/1.73 m^2^ according to a modified Modification of Diet in Renal Disease Study equation [11], and (4) proteinuria ≥1 g/24 h on at least two consecutive laboratory measurements. Exclusion criteria included (1) rapid progressive IgAN (renal function deteriorate quickly or historically necrotic capillaritis and cellular crescents), (2) secondary IgAN such as Henoch-Schönlein purpura, lupus nephritis, and other primary glomerulonephritis, (3) treatment with steroids or cytotoxic drugs during the previous 6 months, (4) pregnancy or planning for pregnancy, and (5) diabetes mellitus, neoplasia, active peptic ulcer disease, viral hepatitis, or other infections. Age- and sex-matched 36 healthy volunteers were concurrently enrolled as controls. 

After a screening assessment, all patients entered a 4-week run-in phase in which eGFR and 24-hour proteinuria were evaluated every 2 weeks. Before enrollment, 28 patients (36.84%) had been treated with ACE inhibitors (ACEI) or angiotensin II receptor blockers (ARB). These patients were required to withdraw the drug at least 4 weeks before eGFR and proteinuria were evaluated. Other antihypertensive agents were allowed to achieve blood pressure target (125/75 mmHg). At the end of the run-in phase, all eligible patients were given oral prednisone at an initial dosage of 1 mg/kg/d for the first 2 months, and gradually tapered by 5 mg till 20 mg/d, then tapered by 2.5 mg to a maintained dosage at 10 mg/d over the next 6 months. Other medication was given simultaneously including (1) an antiplatelet agent, regularly aspirin or change to dipyridamole if the patient has a high risk of bleeding complication, (2) ACEI or ARB, and (3) other antihypertensive agents that could be used to reach the blood pressure target. During the study, the dosage of ACEI or ARB would not change.

### 2.2. Histopathology of IgAN

Paraffin sections were stained with hematoxylin and eosin, periodic acid-Schiff, trichrome, and silver for light microscopy. All histopathologic samples were reviewed and scored independent of previous pathology reviews and by 2 independent renal pathologists who were blinded to previous pathology reviews (in the context of usual clinical care) and patients' clinical outcomes. The light microscopy features of the renal biopsy were examined to evaluate the glomerular changes, which were classified into grades I–V according to the disease severity as described previously [10]. Global and segmental sclerosis was calculated as percentages of the total number of glomeruli (GSS). For tubulo-interstitial lesions, tubular atrophy, interstitial fibrosis, and interstitial inflammatory cell infiltration were scored 0 as absent, 1+ as mild (involving <25% of the interstitium and tubules), 2+ as moderate (involving 25–50% of the interstitium and tubules), and 3+ as intense (involving >50% of the interstitium and tubules), then added up to obtain a final score, tubulo-interstitial damage (TID) score, from 0 to 9 [12]. 

### 2.3. Clinical Parameters

In the morning, after subjects had fasted overnight, blood pressure was measured in the right arm at least twice with a mercury sphygmomanometer after subjects had rested in the supine position for at ≥5 min. Urine and blood samples were collected and analyzed for biochemistry measurements. Serum concentrations of albumin, creatinine, lipids, and hemoglobin (Hb) were measured by a Bayer ADVIA 1650 biochemical instrument. Blood samples were collected and centrifuged at 1500 rpm for 15 min. Serum were collected and stored at −80°C until analyses. 

### 2.4. Followup and Outcome Definition

Patients were examined at baseline, every month for the first 6 months, and then every 3 months. At each visit, body weight, height, blood pressure, serum biochemistry measurement, and 24-hour urine protein excretion were measured and recorded. 

The primary outcome was time to the composite of the first of (1) doubling of baseline serum creatinine, (2) ESRD (permanent hemodialysis, peritoneal dialysis, or renal transplantation), or (3) death from any cause. Treatment outcome was defined as follows: (1) complete remission (CR), which means urinary protein excretion <0.3 g/24 h on 3 consecutive measurements, serum albumin >35 g/L, and renal function stable. (2) Partial remission (PR), which means >50% decrease in urinary protein excretion but >0.3 g/24 h, serum albumin >30 g/L, and renal function stable. (3) Not remission (NR), which means <50% decrease in urinary protein excretion, or deterioration of renal function but <50% decrease in eGFR or less than doubling of Scr.

### 2.5. Analytical Procedure of Serum IL-18 Levels

Serum IL-18 levels were measured using a commercially available enzyme-linked immunosorbent assay kit (Medical and Biologic Laboratories, Nagoya, Japan) that specifically detects the mature form of IL-18, as previously described [13]. The coefficient of variation of inter- and intraassay reproducibility for IL-18 concentration ranges from 5 to 10%, corresponding to that reported by the kit manufacturer. The measurements were made in duplicate and in a blinded fashion. Final serum IL-18 values were expressed in pg/mL. 

### 2.6. Statistical Analysis

Baseline and outcome data were presented as mean ± SD or median (percentual frequency) as appropriate and analysed for significant differences using paired and unpaired *t*-tests for continuous variables (after check for normal distribution), the chi-square test, and Fisher's exact test for categorical variables. The Mann-Whitney *U*-test and Wilcoxon signed-rank test were used for paired and unpaired subjects, respectively. Correlation between serum IL-18 levels and clinical variables were assessed by Spearman correlation coefficient.

The ability of serum IL-18 to discriminate between mild and moderate to severe tubulo-interstitial damage during the follow-up period was determined using receiver operating characteristic (ROC) curves, providing not only sensitivity and specificity, but also the area under the curve (AUC) at different cutoff values of serum IL-18. Kaplan-Meier analysis and logistic regression analysis were used to explore the value for serum IL-18 levels in predicting renal prognosis. Adjusted effects of serum IL-18 levels were presented as odds ratios (OR) with 95% confidence intervals (CI). Two-tailed *P* less than 0.05 is considered statistically significant. Analyses were conducted using SPSS 13.0 software program (SPSS, Incorporated, Chicago, IL, USA).

## 3. Results

### 3.1. Baseline Characteristics of the Study Population

**Figure 1:**
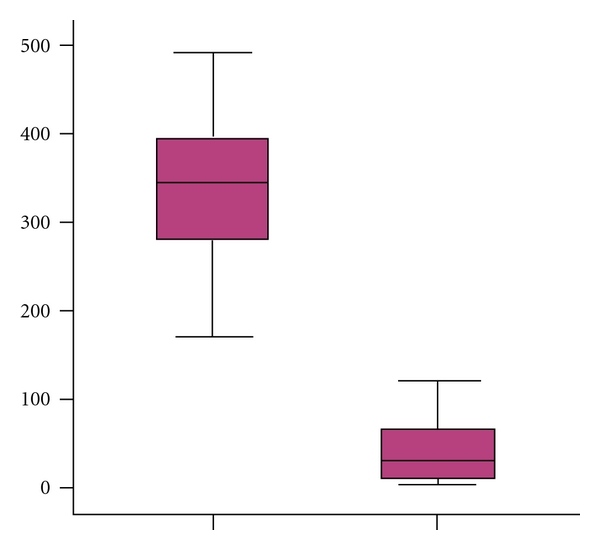
Serum IL-18 concentration was significantly elevated in patients with IgAN than healthy controls (*P* < 0.01).

A total of 76 patients were collected. The patients ranged from 24 to 65 years of age (mean 38.85 ± 10.95 years old), and all presented with proteinuria with a baseline of 2.61 (1.43−4.08) g/24 h (Table 1). The distribution in Lee's grading system of 76 patients was grade III, 17 (22.36%); grade IV, 39 (51.31%); grade V, 20 (26.32%). Compared with healthy controls, baseline serum IL-18 levels were significantly elevated in IgAN patients (360.26 ± 25.23 versus 51.22 ± 8.90 pg/mL, *P* < 0.01, see in Figure 1).

### 3.2. Serum IL-18 Levels after Treatment and Their Correlation with Responsiveness to Corticosteroid in IgAN Patients

**Figure 2:**
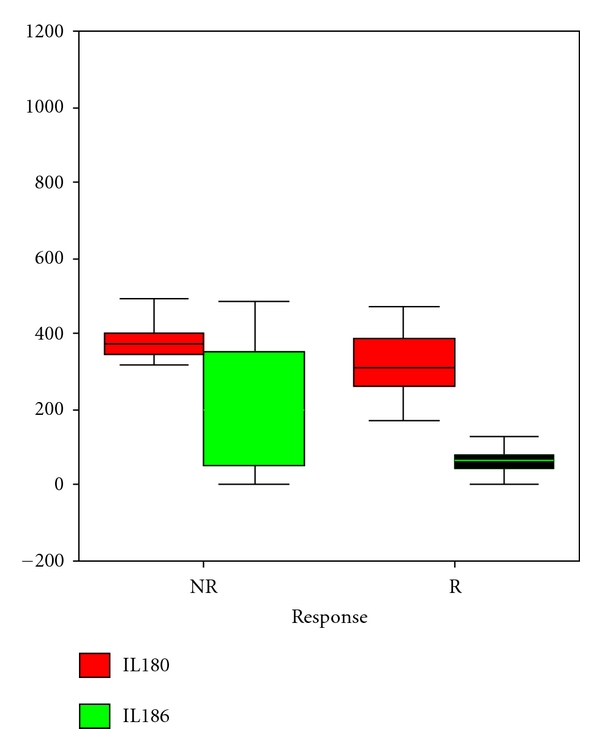
In patients respond to corticosteroid therapy (R group), sIL-18 decreased significantly both in responders and nonresponders (*P* < 0.05) while NRs patients showed much higher baseline IL-18 levels (*P* = 0.02).

After corticosteroid therapy, 29 patients showed CR and 22 patients showed PR, totally 51 patients were deemed responders (R) group (effective rate 67.10%). Those who showed NR to steroid were deemed non-responders (NR). The clinical and histological characteristics of the R and NR patients at the time of enrollment are shown in Table 2. There were no differences between the two groups with respect to age, gender, blood pressure, serum albumin, lipids, hemoglobin, sIgA, renal function, and GGS, whereas NRs showed higher TID scores than Rs (*P* = 0.04). After 12 months therapy, serum IL-18 levels decreased significantly both in the Rs (*P* < 0.01) and NRs (*P* = 0.01) (Figure 2), while NRs patients showed much higher baseline IL-18 levels (384.06 ± 15.10 versus 348.35 ± 37.05, *P* = 0.02). Multivariate regression analysis model which introduces all clinical and histological parameters showed that serum IL-18 levels (*β* = −0.003, *P* = 0.01), serum albumin level (*β* = 0.469, *P* = 0.04), and TID scores (*β* = −0.236, *P* = 0.018) were significantly correlated with corticosteroid responsiveness (Table 3). 

### 3.3. Correlation between Serum IL-18 Levels with Clinical and Histological Parameters

Univariate analysis showed that baseline serum IL-18 levels were siginificantly correlated with sAlb (*r* = −0.395, *P* = 0.001), urinary protein excretion (*r* = 0.494, *P* = 0.002), Scr (*r* = 0.61, *P* < 0.001), and eGFR (*r* = −0.598, *P* < 0.001). With respect to histological parameters, TID scores showed a borderline significance with serum IL-18 levels (*r* = 0.355, *P* = 0.05) whereas GGS did not.

According to proteinuria levels exceeded 3.5 g/24 h or not, we divided our patients into two group. In those who had higher levels of proteinuria, baseline albumin level was significantly decreased (35.42 ± 8.51 versus 38.21 ± 3.66, *P* = 0.02) while serum IL-18 level (402.94 ± 19.86 versus 346.03 ± 15.52, *P* = 0.02) and percentage of glomerular and segmental sclerosis (0.35(0.06–0.47) versus 0.24(0.14–0.26), *P* = 0.05) were significantly increased than those with mild proteinuria (see Table 4). 

**Figure 3:**
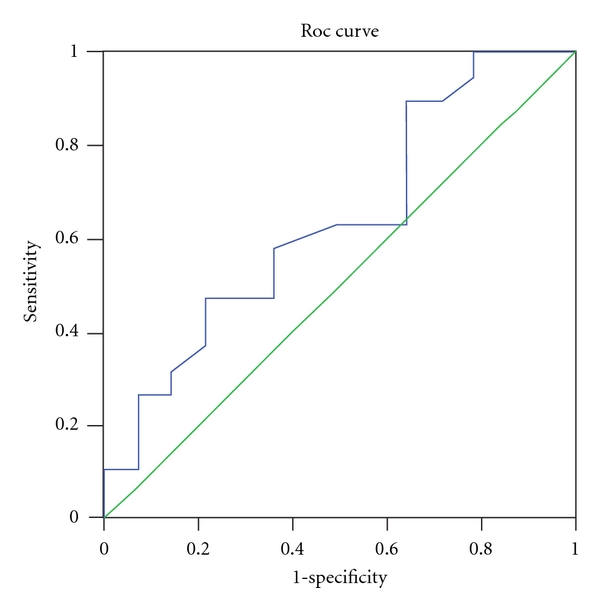
ROC curve for the utility of serum interleukin 18 levels for prediction of tubulo-interstitial damage in IgA nephropathy patients.

We further divided our patients into two groups according to their TID scores. We found that those with severe tubulo-interstitial damage had significantly higher serum IL-18 levels than patients with mild to moderate lesion (367.83 ± 66.83 versus 315.91 ± 65.70, *P* < 0.05). The AUC-ROCs for the utility of sIL-18 for prediction of tubulo-interstitial damage were 0.64 (95% CI: 0.45 to 0.83) (Figure 3). The cutoff value is 323.69 pg/mL, its sensitivity is 94.7%, and specificity is 78.6%, respectively.

### 3.4. Serum IL-18 Levels Predict Reduction of Renal Function in Follow-Up Period

Patients enrolled had a median of follow-up time of 58 (19–120) months. During this period, 4 patients (5.26%) developed the composite primary outcome, 1 patient started hemodialysis, 2 patients started peritoneal dialysis, and 1 patient did renal transplantation all because of ESRD. In addition, another 22 patients (28.95%) had renal function deterioration during followup, whose renal function estimated by GFR decreased at an average of 0.52 ± 0.16 mL/min/1.73 m^2^/month.

Patients were divided into two groups based on whether the renal function decreased or not. The clinical and histological characteristics of the different outcome patients at the time of enrolment are shown in Table 5. Patients who had deteriorated renal function showed higher TID scores (4.00 (3.00−6.00) versus 2.50 (2.00−4.00), *P* = 0.03), higher IL-18 levels (before treatment 364.45 ± 40.25 versus 353.67 ± 16.36, *P* = 0.02; after treatment 132.44 ± 32.40 versus 99.41 ± 24.14, *P* = 0.04), higher Scr (97.50 (71.20–111.80) versus 83.10 (71.00–109.70), *P* = 0.03), lower hemoglobin level (119.00 ± 21.68 versus 128.79 ± 12.45, *P* = 0.03), and lower GFR (71.49 ± 5.11 versus 82.65 ± 7.89, *P* = 0.04) than those who had stable renal function at baseline. Besides, patients who had deteriorated renal function were more likely to be nonresponsive after corticosteroid therapy.

**Figure 4:**
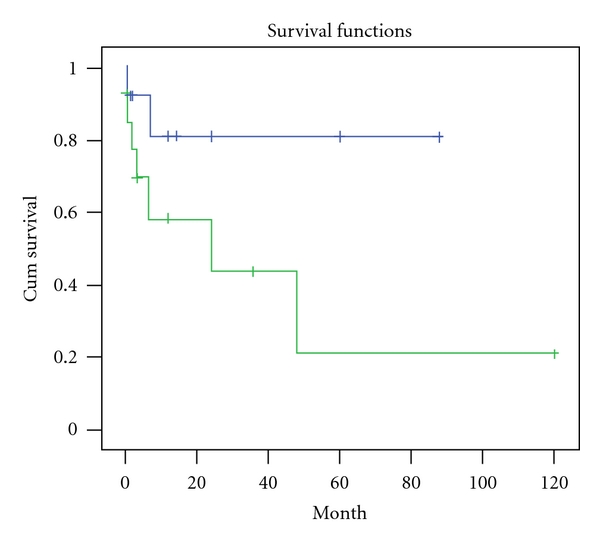
IgAN patients who had higher than median IL-18 levels (346.80 pg/mL) at baseline had worse renal outcome in follow-up period (Log rank *P* = 0.03). In this figure, green line represents those patients with higher sIL-18 levels.

**Figure 5:**
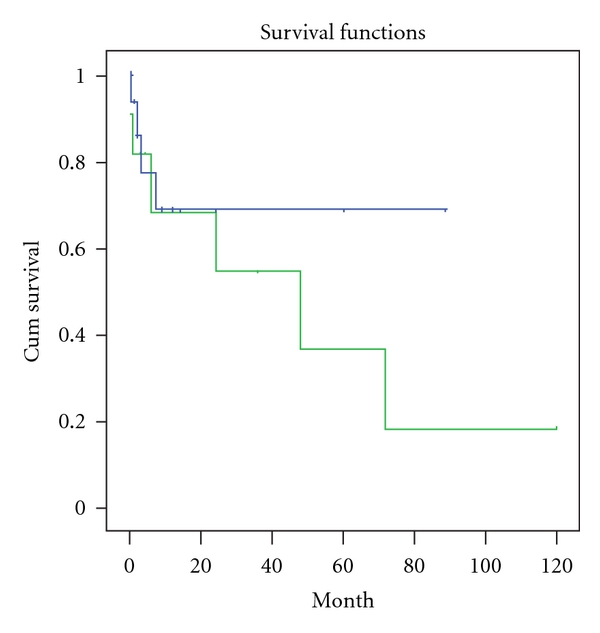
IgAN patients who had higher TID scores at baseline had worse renal outcome in follow-up period (Log rank *P* = 0.005). In this figure, green line represents those patients with higher TID scores.

Univariate analysis found that baseline IL-18 levels (*r* = 0.242, *P* = 0.021), TID scores (*r* = 0.399, *P* = 0.032), Scr (*r* = 0.466, *P* = 0.011), and eGFR (*r* = −0.455, *P* = 0.013) were significantly correlated with the rate of loss of eGFR. The results from the Kaplan-Meier analysis for overall renal survival are shown in Figures 4 and 5. We found that patients who had higher than median IL-18 levels (*P* log rank= 0.03) at baseline and those who had severe tubulo-interstitial damage (*P* log rank=0.005) had worse renal outcome. Baseline serum IL-18 (*β* = 1.98, *P* = 0.003), TID scores (*β* = 1.96, *P* = 0.001), and renal function remained independently associated with the deteriorated renal outcome in Cox proportional hazard model that adjusted for age, smoking and history, blood pressure, albumin, lipids, CRP, and hemoglobin at enrollment (Table 6).

## 4. Discussion

IgAN is the most common form of glomerulonephritis worldwide, in which nearly 40% of cases can lead to ESRD in a chronic and progressive process. As its pathogenesis has not been fully explained, predicting the clinical course has been difficult, and few treatments have demonstrated a significant reduction in progressive disease. Accurately judging which individuals will go on to develop progressive disease would allow physicians to target high-risk patients for aggressive treatment or monitoring. However, physicians have been unable to readily predict individual responses until patients finish a course of immunosuppressive treatment nowadays. As a result, molecular biomarkers may be useful in the diagnosis and prognosis of IgAN in the future.

In this study, we demonstrated that serum IL-18 independently predicts the renal function deterioration in IgAN patients even after adjustment for known clinical predictors. Sensitivity analyses in this relatively small group of patients suggest that IL-18 may be a specific biomarker to be used to evaluate the extent of tubulo-interstitial damage, and even a predictor for disease progression. To the best of our knowledge, this is the first demonstration that IL-18 is an important predictor of patient renal outcome in an IgAN population.

IL-18 is primarily a macrophage-derived cytokine; however, its expression has been reported in a wide range of cells, including those of bone marrow (BM) origin (macrophages, dendritic cells, T cells, and B cells) and parenchymal kidney cells (tubular epithelial cells, podocytes, and mesangial cells) [14, 15]. Recent studies have suggested that IL-18 may provide substantial prognostic information in different settings, especially in tubular injury. IL-18 was found to potentiate ischemic AKI and to be detectable in the urine of mice subjected to ischemic kidney injury [6]. Urinary IL-18 has been studied by Parikh and coworkers in a variety of clinical settings, including delayed graft function [16], cardiac surgery, acute respiratory distress syndrome [17], and cross-sectionally in patients with and without kidney disease [18]. Urinary IL-18 has also been studied as a biomarker of contrast nephropathy [19].

It is also described that patients with CKD, especially those who had a decrease in GFR, have higher serum concentrations of IL-18 than the general population [20–22]. Calvani et al. [23] reported that increased glomerular IL-18 expression in a limited number of renal biopsy specimens from patients with WHO class IV and V lupus nephritis. Hewins et al. [24] reported that IL-18 is upregulated in podocytes, interstitial myofibroblasts, infiltrating interstitial macrophages, and distal tubular epithelial cells in the kidney during active ANCA-associated vasculitis. Tubular IL-18 expression is upregulated in murine models of lupus nephritis and renal ischaemia [6, 25].

While in primary IgAN patients, the relationship between IL-18 with renal pathology and prognosis has not been investigated. We supposed that the mechanism of how elevated IL-18 concentrations predict renal function deterioration may be attributed to its close relationship with tubulo-interstitial injury potentiated by inflammation. Glomerulotubular crosstalk may participate in the development of tubulo-interstitial injury in IgAN [26]. Mediators (mainly TNF-*α*) released from mesangial cells after IgA deposition activate tubular epithelial cell (TEC) and lead to subsequent inflammatory changes in the renal interstitium. The interaction of IgA with glomerular mesangial cells induces cell proliferation [27] and the release of cytokines and chemokines. Activated mesangial cells produce cytokines and chemokines, including IL-1, IL-6, TNF-*α*, monocyte chemotactic protein-1, TGF-*β*, and PDGF [28]. We speculate that these humoral factors/mediators from mesangial cells first activate the podocytes before reaching the tubulointerstitium either by glomerular filtration or by transportation via the postglomerular capillaries. Upon reaching the tubular compartment, these mediators could stimulate TEC to produce other proinflammatory cytokines such as IL-18 and chemokines that eventually lead to tubular damage, interstitial mononuclear cell infiltration, and fibrosis via a multitude of mechanisms. IL-18 plays a crucial role in inflammation and in particular modulates the activity of macrophages [29] by activation of transcription factors including NF-*κ*B [30] and AP-1 [31], inducing transcription of a cascade of inflammatory molecules including inducible nitric oxide synthase (iNOS) [32], TNF-*α* [33], chemokines [34], and adhesion molecules [35]; therefore, IL-18 may promote immune or nonimmune-mediated tissue damage *via *a multitude of mechanisms.

There were several limitations in the present study. First, it was a single-center trial with a small study population carried out over a relatively short period of time. The follow-up time is relatively short to observe the renal outcome. It is suggested that an open-labeled, prospective, multicentered and controlled research is necessary to estimate the value of IL-18 measurement in IgAN and reduce the bias in ours. Secondly, it is an observational study which cannot fully explain why there is a relationship between IL-18 and renal function deterioration in IgAN patients. It provides a clue for us to further investigate the mechanism of this phenomenon and immunosuppressant's effect. Furthermore, it is notable that an international panel of experts recently produced guidelines on the reporting of biopsies that demonstrate IgAN. The Oxford classification system suggests that four features are relevant: (1) mesangial hypercellularity, and(2) segmental sclerosis, (3) endocapillary hypercellularity, and (4) tubular atrophy/interstitial fibrosis. Our study supports the importance of interstitial fibrosis, and glomerular or segmental sclerosis, although we did not recruit other two features. The differences between these results highlight the need for further large, independent samples to confirm the relative importance of inflammation biomarker and other histopathologic features in addition to clinical features.

In conclusion, simple histopathologic measures of tubulo-interstitial injury and serum IL-18 may improve the identification of IgAN patients who are at high risk and have potential for progressive loss of kidney function. These results require confirmation and validation in an independent cohort of patients with biopsy-proven IgAN. We suggest that higher serum IL-18 may represent an ongoing inflammatory and fibrotic process in IgA nephropathy, which indicates for intensive therapy from onset.

## Figures and Tables

**Table 1:** Demographic, clinical, and histological data in IgAN patients at baseline.

Parameter	Data
Male/female	37/39
Age (year)	38.85 ± 10.95
Smoking history (%)	13 (17.11)
SBP (mmHg)	124.42 ± 19.18
DBP (mmHg)	80.58 ± 12.40
Hemoglobin (g/dL)	124.16 ± 18.74
Proteinuria (g/24 h)	2.61 (1.43~4.08)
sAlb (g/L)	36.12 ± 6.26
Scr (**μ**mol/L)	95.90 (78.00~118.40)
eGFR (mL/min/1.73 m^2^)	75.83 ± 4.41
sIgA (mmol/L)	3.09 ± 1.15
Hs-CRP (mg/L)	4.56 ± 0.92
Cholesterol (mmol/L)	5.87 ± 1.24
Triglyceride (mmol/L)	2.68 ± 0.61
sIL-18 (pg/mL)	360.26 ± 25.23
Lee's grading system	
Grade III	17 (22.36%)
Grade IV	39 (51.31%)
Grade V	20 (26.32%)
GSS	0.24 (0.09−0.50)
TID	4.00 (2.00−6.00)

Data are mean ± SD or median interquartile range, and comparisons between groups were made by the Student's *t*-test or *χ*2 test as appropriate. SBP, systolic blood pressure; DBP, diastolic blood pressure; SAlb, serum albumin; Scr, serum creatinine; eGFR, estimated glomerular filtration rate; sIgA, serum immunoglobulin A; Hs-CRP, high-sensitivity C-reactive protein; sIL-18, serum interleukin-18; GSS, global and segmental sclerosis; TID, tubulointerstitial damage.

**Table 2:** Clinical and histological data in Rs and NRs patients.

	Rs patients (*n* = 51)	NRs patients (*n* = 25)	*P* value
Age (y)	39.54 ± 11.33	37.58 ± 10.32	0.61
Female, *n* (%)	23 (45.10%)	12 (50.00%)	0.82
Smokers, *n* (%)	9 (17.64%)	4 (16.00%)	0.58
SBP (mmHg)	124.4 ± 19.2	121.7 ± 15.6	0.06
DBP (mmHg)	80.6 ± 12.4	77.5 ± 7.5	1.35
Hemoglobin (g/L)	125.17 ± 21.17	134.58 ± 8.98	0.07
Albumin (g/L)	38.03 ± 5.99	36.52 ± 5.61	0.35
Scr (*μ*mol/L)	101.32 ± 36.56	94.73 ± 32.39	0.60
eGFR (mL/min/1.73 m^2^)	73.32 ± 25.27	80.85 ± 29.09	0.45
Urinary protein (g/24 h)	2.73 (1.65–3.91)	1.86 (1.28–2.69)	0.13
sIgA (g/L)	3.05 ± 1.07	3.38 ± 0.68	0.33
sIL-18 (pg/mL)	348.35 ± 37.05	384.06 ± 15.10	0.02
GGS (%)	0.22 (0.09–0.43)	0.25 (0.08–0.50)	0.67
TID	3.00 (2.00–4.00)	4.50 (3.00–6.00)	0.04

**Table 3:** Multivariate regression model to evaluate correlated factors with responsiveness to steroid therapy.

Parameters	*β*	*P* value
Sex	0.112	0.559
Age (year)	0.023	0.899
Smoke duration	−0.362	0.238
SBP (mmHg)	0.279	0.112
DBP (mmHg)	0.022	0.099
Hemoglobin (g/dL)	−0.057	0.761
Proteinuria (g/24 h)	0.070	0.704
sAlb (g/L)	0.469	0.040
Scr (**μ**mol/L)	0.157	0.384
eGFR (mL/min/1.73 m^2^)	−0.195	0.278
sIgA (mmol/L)	−0.104	0.570
Hs-CRP (mg/L)	−0.078	0.474
Cholesterol (mmol/L)	−0.638	0.881
Triglyceride (mmol/L)	−0.294	0.729
sIL-18 (pg/mL)	−0.003	0.010
Lee's grading system	−0.075	0.676
GSS	−0.151	0.398
TID	−0.236	0.018

**Table 4:** Clinical and histological data between patients with proteinuria above 3.5 g/24 h or not.

	Proteinuria lower than 3.5 g/24 h (*n* = 57)	Proteinuria above 3.5 g/24 h (*n* = 19)	*P* value
Age (y)	38.87 ± 10.76	37.49 ± 9.98	0.88
Female, *n* (%)	28 (49.12%)	11 (57.89%)	0.67
Smokers, *n* (%)	10 (17.54%)	3 (15.79%)	0.49
SBP (mmHg)	124.54 ± 18.33	118.8 ± 14.02	0.52
DBP (mmHg)	79.62 ± 10.90	77.24 ± 7.91	0.56
Hemoglobin (g/L)	131.15 ± 19.38	119.78 ± 14.04	0.15
Albumin (g/L)	38.21 ± 3.66	35.42 ± 8.51	0.02
Scr (*μ*mol/L)	100.32 ± 35.42	95.37 ± 35.09	0.99
eGFR (mL/min/1.73 m^2^)	72.89 ± 25.40	80.64 ± 29.00	0.37
sIgA (g/L)	3.13 ± 0.97	3.26 ± 1.00	0.62
sIL-18 (pg/mL)	346.03 ± 15.52	402.94 ± 19.86	0.02
GGS (%)	0.24 (0.14–0.26)	0.35 (0.06–0.47)	0.05
TID	4.00 (3.00–4.00)	4.50 (2.00–6.00)	0.88

**Table 5:** Comparison of clinical and histological parameters between IgAN patients with renal function deterioration or not in follow-up.

Parameters	Renal function deteriorated (*n* = 26)	Renal function stable (*n* = 50)	*P* value
Age (year)	39.71 ± 9.59	37.12 ± 11.07	0.38
SBP (mmHg)	130.02 ± 20.18	125.42 ± 18.09	0.27
DBP (mmHg)	85.03 ± 10.40	80.00 ± 13.91	0.19
Hemoglobin (g/dL)	119.00 ± 21.68	128.79 ± 12.45	0.03
Proteinuria (g/24 h)	2.4 (1.4~4.0)	2.6 (1.7~3.1)	0.56
sAlb (g/L)	36.1 ± 6.2	38.9 ± 3.9	0.34
Cholesterol (mmol/L)	5.78 ± 1.36	5.90 ± 1.19	0.89
Triglyceride (mmol/L)	2.49 ± 0.70	2.78 ± 0.58	0.08
Scr (**μ**mol/L)	97.50 (71.20–111.80)	83.10 (71.00–109.70)	0.03
eGFR (mL/min)	71.49 ± 5.11	82.65 ± 7.89	0.04
sIgA (g/L)	3.2 ± 0.7	3.1 ± 1.2	0.28
Hs-CRP (mg/L)	4.76 ± 1.01	4.34 ± 0.98	0.30
sIL-18 at baseline (pg/mL)	364.45 ± 40.25	353.67 ± 16.36	0.02
sIL-18 after treatment (pg/mL)	132.44 ± 32.40	99.41 ± 24.14	0.04
GSS	0.28 (0.09–0.50)	0.25 (0.08–0.43)	0.78
TID	4.00 (3.00–6.00)	2.50 (2.00–4.00)	0.03
Steroid responsiveness (*n*)	19	32	0.06

**Table 6:** Cox Regression analysis using to evaluate risk factor for renal function deterioration in patients with IgA nephropathy.

Parameter	*β*	*t*	*P* value
TID score	1.96	3.69	0.001
sIL-18 (pg/mL)	1.98	2.77	0.003
Baseline Scr (*μ*mol/L)	0.09	0.19	0.017
eGFR (mL/min/1.73 m^2^)	−0.05	0.01	0.023
Proteinuia (g/24 h)	2.50	0.33	0.056
Gender	0.56	0.32	0.572
Age (year)	−0.19	−3.98	0.312
Smoke history	0.33	0.41	0.746
SBP (mmHg)	−0.03	−0.27	0.760
DBP (mmHg)	0.02	0.23	0.821
Hemoglobin (g/dL)	0.22	1.43	0.739
sAlb (g/L)	0.002	0.02	0.734
Cholesterol (mmol/L)	0.07	0.59	0.653
Triglyceride (mmol/L)	−0.18	−1.53	0.533
sIgA (g/L)	0.18	1.97	0.812
Hs-CRP (mg/L)	0.13	1.45	0.931
GSS (%)	−0.04	−0.36	0.602
